# Artificial intelligence-based models for quantification of intra-pancreatic fat deposition and their clinical relevance: a systematic review of imaging studies

**DOI:** 10.1007/s00330-025-11808-6

**Published:** 2025-07-19

**Authors:** Tej Joshi, John Virostko, Maxim S. Petrov

**Affiliations:** 1 School of Medicine, University of Auckland, Auckland, New Zealand; 2 Department of Diagnostic Medicine, Dell Medical School, University of Texas at Austin, Austin, TX USA; 3 Livestrong Cancer Institutes, Dell Medical School, University of Texas at Austin, Austin, TX USA; 4 Department of Oncology, Dell Medical School, University of Texas at Austin, Austin, TX USA; 5 Oden Institute for Computational Engineering and Sciences, University of Texas at Austin, Austin, TX USA

**Keywords:** Artificial intelligence, Body fat distribution, Diabetes mellitus, Pancreatic neoplasms, Pancreatitis

## Abstract

**Abstract:**

High intra-pancreatic fat deposition (IPFD) plays an important role in diseases of the pancreas. The intricate anatomy of the pancreas and the surrounding structures has historically made IPFD quantification a challenging measurement to make accurately on radiological images. To take on the challenge, automated IPFD quantification methods using artificial intelligence (AI) have recently been deployed. The aim was to benchmark the current knowledge on the use of AI-based models to measure IPFD automatedly. The search was conducted in the MEDLINE, Embase, Scopus, and IEEE Xplore databases. Studies were eligible if they used AI for both segmentation of the pancreas and quantification of IPFD. The ground truth was manual segmentation by radiologists. When possible, data were pooled statistically using a random-effects model. A total of 12 studies (10 cross-sectional and 2 longitudinal) encompassing more than 50 thousand people were included. Eight of the 12 studies used MRI, whereas four studies employed CT. U-Net model and nnU-Net model were the most frequently used AI-based models. The pooled Dice similarity coefficient of AI-based models in quantifying IPFD was 82.3% (95% confidence interval, 73.5 to 91.1%). The clinical application of AI-based models showed the relevance of high IPFD to acute pancreatitis, pancreatic cancer, and type 2 diabetes mellitus. Current AI-based models for IPFD quantification are suboptimal, as the dissimilarity between AI-based and manual quantification of IPFD is not negligible. Future advancements in fully automated measurements of IPFD will accelerate the accumulation of robust, large-scale evidence on the role of high IPFD in pancreatic diseases.

**Key Points:**

***Question*** *What is the current evidence on the performance and clinical applicability of artificial intelligence-based models for automated quantification of intra-pancreatic fat deposition?*

***Findings*** *The nnU-Net model achieved the highest Dice similarity coefficient among MRI-based studies, whereas the nnTransfer model demonstrated the highest Dice similarity coefficient in CT-based studies.*

***Clinical relevance*** *Standardisation of reporting on artificial intelligence-based models for the quantification of intra-pancreatic fat deposition will be essential to enhancing the clinical applicability and reliability of artificial intelligence in imaging patients with diseases of the pancreas.*

## Introduction

High intra-pancreatic fat deposition (IPFD) is the most common pathology of the pancreas, surpassing type 2 diabetes mellitus (T2DM) and acute pancreatitis in frequency [1]. Moreover, high IPFD is also associated with incident pancreatic cancer and chronic pancreatitis [2]. While it necessitates precise diagnostic and monitoring tools, accurate measurement of intra-pancreatic fat deposition (IPFD) is challenging due to the anatomical and physiological characteristics of the pancreas. Specifically, the pancreas is located deep in the retroperitoneum and has an elongated serpiginous shape. Furthermore, its embedding in visceral fat (that may invaginate between pancreatic lobules) can further complicate IPFD quantification and make intra-pancreatic and peri-pancreatic fat differentiation more challenging. Since invasive approaches (such as surgery or endoscopic ultrasound-guided fine needle biopsy) would be unethical in most cases and would carry the risk of adverse effects, IPFD quantification predominantly relies upon non-invasive imaging modalities (such as CT and chemical shift-encoded MRI) [1].

The most frequently used modern approach to quantification of IPFD is manual segmentation of the pancreas and subsequent quantification of the fat within the region of interest (defined within the borders of the organ) [3, 4]. However, manual segmentation of the pancreas requires considerable expertise, labour, and time. Given the already large workload that radiologists typically face nowadays, the additional burden of that task would likely be overwhelming. Automated pancreas segmentation and quantification of IPFD using artificial intelligence (AI) algorithms has the potential to meet the challenge [5]. AI is a rapidly growing field, thanks to recent increases in the quantity of data as well as computational power. AI broadly refers to methods and algorithms that mimic human intelligence for problem-solving and learning tasks [6]. Machine learning and deep learning (DL) are nested subsets of AI that have recently gained traction in medical imaging for their use in organ segmentation. Machine learning encompasses a class of algorithms capable of identifying patterns and relationships within data (such as in segmentation tasks), learning from input data and improving performance through experience [6, 7]. Within machine learning, DL is a specialised subset that utilises artificial neural networks to automatically learn hierarchical patterns from images during a training phase. DL represents a significant advancement through multi-layered neural networks, enabling automated extraction of meaningful features from complex medical imaging datasets [6, 8]. Convolutional Neural Networks (CNNs) are a subset of DL that uses the spatial arrangement within imaging data to learn and later produce outputs [8]. CNNs effectively capture spatial hierarchies within images and are therefore most commonly applied to imaging data (e.g., for segmentation of abdominal organs). The integration of AI in the quantification of IPFD can reduce the manual labour associated with segmentation and potentially enhance the consistency in disease detection and characterisation. As AI has recently made a big leap forward, it is timely to map all available studies of AI-based models for automated quantification of IPFD—a gap in the current imaging literature [9, 10].

The aim of this study was to systematically review imaging studies that used AI-based models to quantify IPFD. Furthermore, we explored the clinical relevance of these models to assess their capacity for clinical adoption.

## Methods

### Search strategy

A systematic literature search was conducted using four electronic databases: Embase, Scopus, MEDLINE, and IEEE Xplore. The search string was as follows: (pancrea*) AND (artificial intelligence OR artificial OR reasoning OR deep learning OR machine learning OR supervised learning OR unsupervised learning OR neural network* OR automat* OR generation adversarial* OR convolution*) AND (fat* OR steatos* OR steatotic OR adipo* OR lip*) AND (imag* OR imaging OR scan* OR ultrasound OR sonograph* OR endoscopic ultrasound OR endoscopic sonograph* OR magnetic resonance imag* OR mri OR computed tomograph* OR ct OR tomograph*) AND (quant* OR quantif* OR volume OR fraction OR percent* OR ratio OR deposi*). The search end date was November 1, 2024. All search results were exported, compiled in the Rayyan software [11], and duplicates were removed. The remaining studies were then screened based on titles and abstracts. Relevant articles were also identified through personal library files. No restrictions were placed on the language of publication.

### Eligibility criteria

Eligible studies were required to use radiological imaging methods (MRI, CT, ultrasound, or endoscopic ultrasound) for assessment of IPFD; studies using biopsy or histological methods, or manual segmentation (unless for establishing ground truth) or radiomics and texture analysis were excluded [12]. Studies on human subjects of any age, sex, ethnicity, and comorbidities were included. Original studies had to be cross-sectional or longitudinal in design; case reports and case series (fewer than 20 participants) were excluded. Studies were eligible if they used AI for both segmentation of the pancreas and automated quantification of IPFD. Studies of automated pancreas segmentation alone (without automation of IPFD quantification) were ineligible [9]. Studies only on the use of non-AI software and algorithms to quantify IPFD after the pancreas has already been segmented (such as proton density fat fraction (PDFF) readouts as part of modern commercially available scanners) were excluded. Furthermore, studies that did not segment the entire pancreas were excluded. Studies on lipomatous pseudohypertrophy or pancreatic lipoma, and those with participants undergoing partial or complete pancreatic resections, were also excluded.

### Study domains

The developmental domain involved studies that used AI to segment the pancreas and automated (non-human) means to quantify IPFD. The technical and analytical aspects of the AI-based models itself (compared with manual segmentation and quantification) were the primary focus of these studies. The clinical domain involved studies that used AI-based models to segment the pancreas and quantify IPFD, with the primary focus on reporting clinical or radiological endpoints (rather than focusing on the comparison between humans versus AI). Eligible studies were categorised into the developmental domain, clinical domain, or both.

### Data extraction

For each eligible study, the following data were extracted: authors, year, country, design, imaging modality (including the scanner used, field strength, imaging sequence, contrast use and phase, preprocessing and postprocessing methods), data source, total number of participants, sex of participants, their age and body mass index (BMI), AI algorithm used for segmentation, IPFD quantification method, segmentation and quantification performance metrics (e.g., Dice similarity coefficient (DSC), Jaccard index, Hausdorff distance), ground truth for segmentation and quantification, and clinical endpoints. The DSC, which was derived from AI/automated segmentation of the whole pancreas and the ground truth (manual segmentation), ranged from 0% (i.e., no overlap) to 100% (i.e., perfect agreement between AI/automated segmentation and ground truth). The Jaccard index was another measure of similarity, defined as the intersection of two sets divided by their union. The Hausdorff distance was a measure of distance between two sets of points in a metric space.

### Methodological quality

The Joanna Briggs Institute’s critical appraisal tool for analytical studies was used to assess the quality of all included studies [13]. This instrument consists of 8 (for cross-sectional studies) and 11 (for longitudinal studies) questions related to features of the study such as the population, setting, exposure, measurement, confounders, outcomes and statistical analysis. Questions can be answered as “Yes”, “No”, “Unclear” or “Not applicable” [13].

### Statistical analysis

Statistical analysis was conducted using the RevMan software version 5.4. The Z-value tested the null hypothesis that the mean effect size is zero. Heterogeneity across studies was assessed using the I² statistic, which measures the percentage of total variation across studies attributable to heterogeneity rather than chance. An I² value of less than 25% was deemed to indicate low heterogeneity, 25% < I^2^ < 75%—moderate heterogeneity, and more than 75%—substantial heterogeneity. A *p*-value of less than 0.05 was deemed statistically significant.

## Results

### Study characteristics

**Fig. 1:**
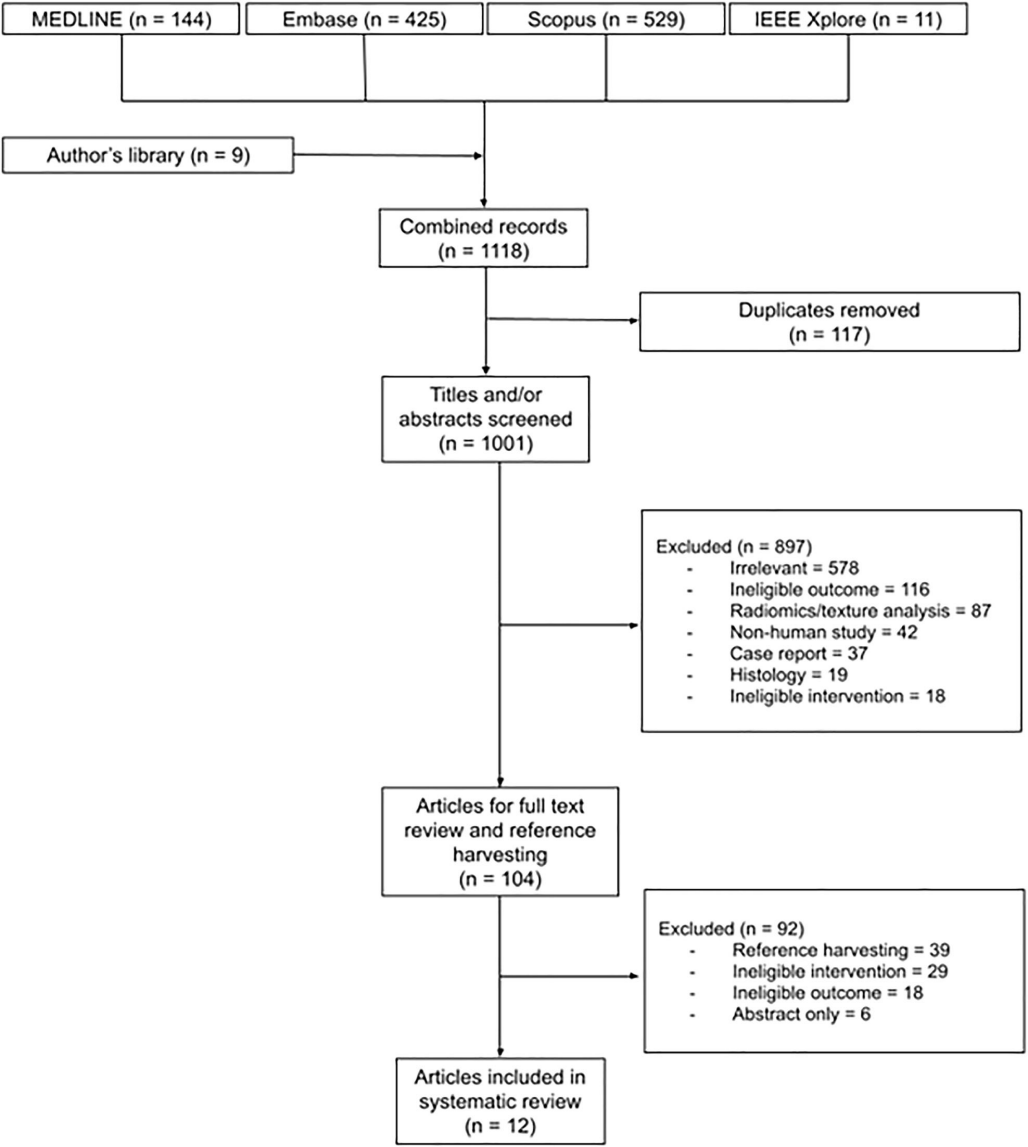
Study selection process

Out of 1001 unique publications identified, 12 studies met all the eligibility criteria (Fig. 1). These studies were published between 2020 and 2024, with the highest percentage of them published in 2022 (*n* = 4, 33.3%) and the lowest percentage of them published in 2020 (*n* = 1, 8.3%). Four studies [14–17] fell under the developmental domain, six [18–23] under the clinical domain, and two [24, 25] belonged in both domains. The overwhelming majority of studies were cross-sectional (*n* = 10, 83.3%), whereas two studies were longitudinal (16.7%) (Table 1). Methodological quality of the included studies is summarised in Supplementary Table 1.

**Table 1:** Characteristics of the included studies

Study ID	Country	Design	Imaging modality	Source of data	Total participants, *n*	Sex (men, women), *n*	Age (years), mean ± SD	BMI (kg/m^2^), mean ± SD	Domain
Basty et al* [14]	United Kingdom	Cross-sectional	MRI	UKBB	98	NR	NR	NR	Developmental
Dong et al [19]	United Kingdom	Longitudinal cohort	MRI	UKBB	42,599	19,855, 22,744	65^a^ (IQR = 58 to 70)	25.7^a^ (IQR = 23.4 to 28.5)	Clinical
Gatidis et al [20]	United Kingdom, Germany	Cross-sectional	MRI	UKBB and NAKO	17,996	9160, 8836	57.3 ± 11.2	26.8 ± 4.5	Clinical
Lin et al [15]	China	Cross-sectional	MRI	NR	176	90, 86	27.3^b^	NR	Developmental
Liu et al [21]	United Kingdom	Cross-sectional	MRI	UKBB	25,617	12,501, 13,116	64.2 ± 7.5	26.5 ± 4.3	Clinical
Tallam et al* [24]	United States	Cross-sectional	CT	Hospital dataset	8992	3983, 5009	57 ± 8	28.9 ± 6.5	Both
Tanabe et al [22]	Japan	Cross-sectional	CT	Hospital dataset	128	80, 48	55.4 ± 20.6	NR	Clinical
Triay Bagur et al [18]	United Kingdom	Cross-sectional	MRI	UKBB	1170	NR	56.7^b^	28.3^b^	Clinical
Whitcher et al [23]	United Kingdom	Longitudinal cohort	MRI	UKBB	3088	1540, 1548	62.9^b^	26.2^b^	Clinical
Yang et al [25]	New Zealand	Cross-sectional	MRI	Hospital dataset	394	249, 145	47.8 ± 1.2	28.8 ± 0.7	Both
Yang et al* [16]	South Korea	Cross-sectional	CT	Hospital dataset	851	104, 115	66.8 ± 9.3	23.0^a^ (IQR = 17.3–33.3)	Developmental
Zhang et al* [17]	China	Cross-sectional	CT	Hospital dataset	227	127, 100	53.5 ± 13.8	NR	Developmental

Country refers to where the study population came from

In the study by Gatidis et al [20], a total of 17,996 participants (8791 from UKBB and 9205 from NAKO) were included

Mean age and BMI are based on the total participants (unless specified otherwise)

In the study by Lin et al [15], the mean was calculated from three provided datasets. While specific BMI data were not reported, the study included 98 individuals with a BMI > 28 kg/m^2^

In the study by Whitcher et al [23], means were provided separately for men and women for age and BMI, from which a combined mean was calculated

The study by Yang et al [16] did not provide mean age and BMI information on the total population, but rather on their external testing set

*BMI* body mass index, *CT* computed tomography, *IQR* interquartile range, *MRI* magnetic resonance imaging, *NAKO* German National Cohort, *NR* not reported, *SD* standard deviation, *UKBB* United Kingdom Biobank

* The study provided data suitable for meta-analysis

^a^ Median

^b^ Mean

Six of the 12 studies [14, 18–21, 23] used the UK Biobank (UKBB), with a possible overlap in study participants. While most cross-sectional studies based on the UKBB [14, 18, 20, 21] did not report on the study periods, one study noted their study period (from July 2014 to January 2023) [19]. The longitudinal study based on the UKBB reported that their participants underwent a first MRI in December 2020, with a maximum follow-up period of 3 years [23].

### Imaging characteristics and AI-based models

Eight of the 12 studies were MRI studies [14, 15, 18–21, 23, 25] and, of these, two studies fell under the developmental domain, five under the clinical domain, and one under both. The remaining four were CT studies [16, 17, 22, 24], with two belonging to the developmental domain, one in the clinical domain, and one in both (Table 2).

**Table 2:** Imaging protocols and computational details of the included studies

Study ID	Scanner	Field strength	Imaging sequence or contrast use and phase	Image preprocessing	Image postprocessing	AI segmentation method	Computation details for AI model training
**MRI**							
Basty et al [14]	NR	1.5 Tesla	Combination of 3D T1-weighted and multi-echo MRI sequences	Normalisation and minor translations	Binarising the network output by thresholding non-zero values, erosion of two voxels to avoid partial volume effects or inclusion of non-pancreatic tissue, and discarding non-contiguous voxels unconnected to the largest segmented object	V-net	CPU: NR GPU: NVIDIA Titan V with 12 GB GPU memory Software environment: NR Training duration: 185 epochs (39 h to convergence) Batch size: one Learning rate: 0.00005
Dong et al [19]	NR	1.5 Tesla	2-point Dixon acquisition sequence	NR	Outermost pixel layer removed to eliminate abnormal hyperintensity due to the partial volume effect	nnU-Net	NR
Gatidis et al [20]	Siemens MAGNETOM Avanto (UK Biobank) Siemens MAGNETOM Skyra (German National Cohort)	1.5 Tesla (UK Biobank) 3.0 Tesla (German National Cohort)	T1-weighted Dixon MRI	NR	NR	nnU-Net	NR
Lin et al [15]	Siemens MAGNETOM Prisma, GE DISCOVERY MR750W, Philips Ingenia Prodiva CX	3.0 Tesla (Siemens and GE platforms), 1.5 Tesla (Philips platform)	3D T1-weighted dual-echo Dixon sequence with gradient echo techniques	Cropping peripheral zero pixel regions, adaptive resampling of images to a unified resolution, and z-transform normalisation of IP/OP images	Outermost pixel layer removed to eliminate abnormal hyperintensity due to the partial volume effect	nnU-Net	CPU: 24 AMD EPYC 7302 16-Core Processors GPU: Five NVIDIA GeForce RTX 3090s Software environment: System running CentOS 7.7 Training duration: 500 epochs Batch size: 250 minibatches (2 patches per minibatch) Learning rate: 0.01 decaying polynomially over training iterations
Liu et al [21]	Siemens Aera	1.5 Tesla	3D T1-weighted dual-echo Dixon sequence	Dixon data combined into 3D volumes with automated correction; no preprocessing of T1w 3D pancreas data	NR	U-Net	NR
Triay Bagur et al [18]	Siemens MAGNETOM Avanto	1.5 Tesla	3D two-point Dixon sequence and a 2D axial multi-echo gradient-recalled echo sequence	NR	NR	U-Net	NR
Whitcher et al [23]	NR	1.5 Tesla	3D T1-weighted dual-echo Dixon sequence	NR	NR	V-Net	NR
Yang et al [25]	Siemens MAGNETOM Skyra	3.0 Tesla	NR	Superpixel segmentation and image dimensionality reduction	Erosion algorithm to remove redundant extra boundaries	U-Net	CPU: Intel i9-11900K GPU: NVIDIA RTX 2080 Ti Software environment: Python 3.6.0 with TensorFlow and Keras deep learning frameworks Training duration: 20 epochs Batch size: NR Training rate: NR
**CT**							
Tallam et al [24]	NR	NA	Non-contrast	Adaptive normalisation and minor translations in imaging dimensions	Morphologic erosions and thresholding	Ensemble of three U-net models	NR*
Tanabe et al [22]	GE Optima CT660 Pro, Siemens SOMATOM Drive	NA	Non-contrast	NR	NR	NR	NR
Yang et al [16]	NR	NA	Contrast-enhanced (portal venous phase)	Normalisation of intensity and voxel size. Intensity values clipped to the 0.5 to 99.5 percentiles and z-normalisation applied using the mean and standard deviation derived from all intensity values	NR	nnU-Net	CPU: NR GPU: Four NVIDIA RTX-A6000s Software environment: TensorFlow 2.6 deep learning framework Training duration: 400 epochs Batch size: four Learning rate: Initially 0.0003 with Adam optimiser
Zhang et al [17]	NR	NA	Contrast-enhanced (portal venous phase)	3D NumPy matrix derived from Nifti or DICOM images of enhanced abdominal CT scans	NR	nnTransfer	CPU: NR GPU: NVIDIA RTX8000 Software environment: Python 3.9 on a Rocky Linux 8.7 system Training duration: 123 epochs Batch size: four Training rate: Initially 0.0004 with AdamW optimiser

*AI* artificial intelligence, *CPU* central processing unit, *CT* computed tomography, *GPU* graphics processing unit, *MRI* magnetic resonance imaging, *NA* not applicable, *NR* not reported

* Specific hardware details were not explicitly mentioned, although the computation resource utilised was the National Institutes of Health Biowulf high-performance computing cluster

Five of the studies in the developmental (or both developmental and clinical) domain [14–17, 25] provided information regarding the computational details of the hardware and software systems used to train their respective AI models (Table 2). These included the central processing unit used, the graphics card used, the software environment, the training duration, the batch size and the learning rate. One study in the developmental domain [24] and the remaining studies in the clinical domain did not include computational details. Studies in the clinical domain used pre-developed AI models for their segmentation tasks.

Four studies employed a U-Net model for their segmentation tasks [18, 21, 24, 25] (33.3%), four—an nnU-Net model [15, 16, 19, 20] (33.3%), two—a V-Net model [14, 23] (16.7%), one—an nnTransfer model [17] (8.3%). One study (8.3%) did not specify the model used [22].

### Developmental domain

**Fig. 2:**
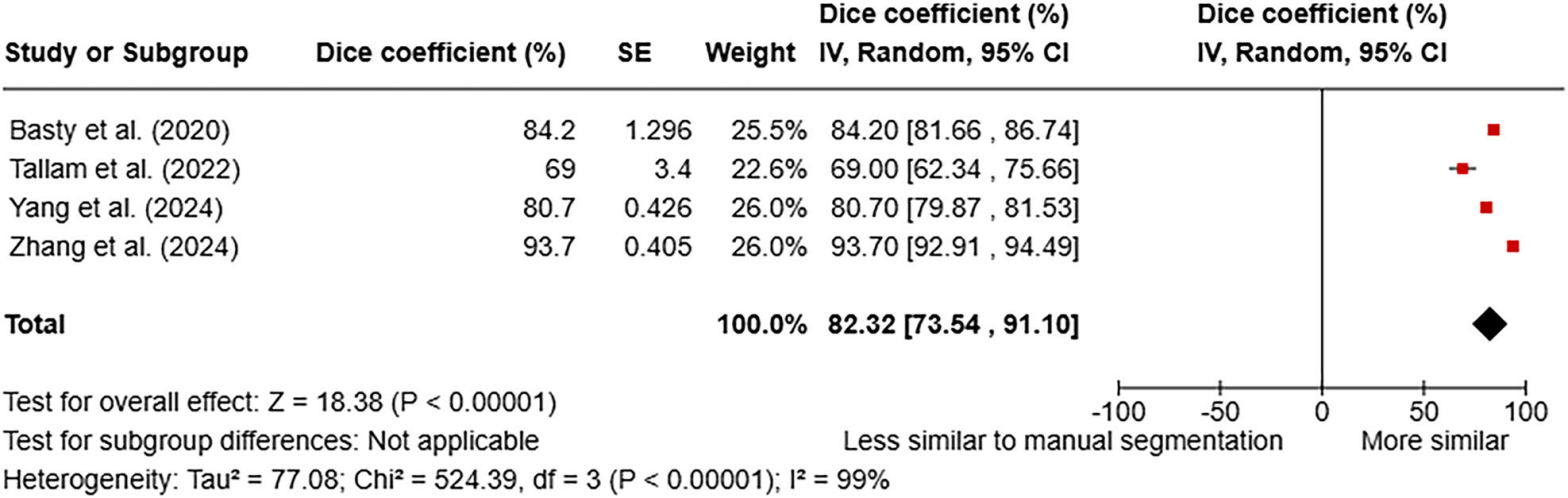
Pooled performance of AI models in quantifying high intra-pancreatic fat deposition

Six studies compared AI-based models with manual reference standards [14–17, 24, 25] (Table 3). There was no uniform metric comparing IPFD quantification methods: metrics such as *R*^2^ [25], ICC [15, 16], and PCC [17] were used. One study calculated a mean difference [24], and one study presented results in a histogram [14]. Four studies suitable for meta-analysis were pooled to determine the performance of AI-based models using the DSC [14, 16, 17, 24]. The meta-analysis yielded a mean pooled DSC of 82.3% (Fig. 2). The Z-value was 18.382 (*p* < 0.001). The I^2^ statistic was 99%, indicating high statistical heterogeneity. All four studies employed different AI-based models (nnTransfer, V-Net, U-Net, and nnU-Net). Three of these were CT studies [16, 17, 24] and one was an MRI study [14].

**Table 3:** Characteristics of artificial intelligence models used for comparison with reference standards

Study ID	AI model	Imaging modality	Segmentation				IPFD quantification			
			Ground truth	Results based on	DSC (%), mean ± SD	Other measures of similarity, means	Manual IPFD quantification method	Automated IPFD quantification method	Results based on	Main findings
**Direct comparison**										
Lin et al [15]	nnU-Net	MRI	Manual segmentation by two radiologists (5 and 3 years of experience)—supervised by a senior radiologist	Internal validation set of 44	91.6	PPV = 0.9195 Sensitivity = 0.9232	Nine ROIs on the pancreas in the FF map. Mean FF was calculated for each subregion and the whole pancreas. Done separately by 3 radiologists.	FF calculated in each voxel in pancreatic masks by fat/(fat + water) × 100%. Mean FF value calculated on each subregion and the whole pancreas. AI segmentations post-processed with erosions to minimise partial volume effects.	Six subjects from the internal validation set	Test-retest reliability for whole pancreas: nnU-Net model ICC = 0.936 (*p* < 0.001) Radiologist A ICC = 0.737 (*p* < 0.05)^a^
Yang et al [25]	U-Net	MRI	Manual segmentation by study authors	Internal validation set of 79	91.2	NR	Manually quantified pancreatic fat pixel area, excluding pixels representing blood vessels, ducts, or visceral fat with fat percentages outside 1–20%.	Configured DCNN to mirror this function, using OpenCV’s erosion algorithm to refine segmented image boundaries.	External validation data (240 images from 10 additional participants)	*R*^2^ = 0.9675
Yang et al [16]	nnU-Net	CT	Manual segmentation by two radiologists (14 and 7 years experience)	External testing set of 219	In the external testing set: DL vs R1: 80.7 ± 6.3^b^	Jaccard Index: DL vs R1: 0.403 ± 0.031	Pancreatic fat volume obtained using the ROI method by two radiologists.	Fat area found automatically within the range of −190 to −15 HU on CT.	External testing set of 219	AI model measured larger fat volume than radiologist (1.2 ± 1.6 cm^3^ vs 0.6 ± 1.3 cm^3^, respectively, *p* < 0.001) ICC: DL vs R1 = 0.73^a^
**Indirect comparison**										
Basty et al [14]	V-Net	MRI	Manual segmentation by radiographer	Internal validation set of 30	84.2 ± 7.1	NR	PDFF value readout from manual and AI segmentations		Internal validation set of 30	Histogram showed that 27 out of 30 median PDFF values for the test data overlapped when comparing PDFF quantification in manual versus AI-derived segmentation
Tallam et al [24]	U-Net	CT	Manual segmentation by trained research assistant, and checked by radiologist (28 years of experience)	25 randomly selected cases from the internal validation set	69 ± 17	Hausdorff distance = 29.63 ± 19.91 mm Jaccard Index = 0.54 ± 0.17 ASSD = 4.57 ± 4.66 mm RAVD = −0.10 ± 0.20	Fraction of voxels between −190 HU and −30 HU. AI segmentations post-processed with erosions to minimise partial volume effects. Pancreatic fat fraction readouts were determined in manual and AI segmentations.		25 randomly selected cases from the internal validation set	Mean difference = −0.0038 ± 0.0108, *p* = 0.09
Zhang et al [17]	nnTransfer	CT	Manual segmentation by one radiologist (> 15 years of experience)	Internal validation set of 22	93.7 ± 1.9	Hausdorff distance = 2.655 ± 1.479 mm	CT attenuation of < −20 HU was used to define IPFD. Comparison of annotated labels by radiologists versus those predicted by model.		Internal validation set of 22	PCC > 0.979 for fat volume, *p* < 0.001

Only studies belonging to the developmental domain are presented. Quantification of IPFD was compared in two ways across studies in this domain: three studies compared manual IPFD quantification (ROI method or other) versus automated IPFD quantification [15, 16, 25], whereas three other studies compared IPFD quantification (a readout from the scanner) in manual versus AI segmentation [14, 17, 24]

The study by Lin et al [15] trained four different nnU-Net models (2D dual-contrast, 3D one-contrast (IP), 3D one-contrast (OP) and 3D dual-contrast). A mean DSC of 91% for the 3D dual-contrast nnU-Net model was found, which outperformed the 2D dual-contrast and 3D one-contrast models. In their study, Yang et al [16] achieved a mean DSC of 76% in the internal validation dataset and a higher DSC of 80% in the external test dataset, when compared with manual segmentations by two radiologists (and the STAPLE algorithm, respectively

*AI* artificial intelligence, *ASSD* average symmetric surface distance, *CT* computed tomography, *DCNN* deep convolutional neural network, *DL* deep learning, *DSC* Dice similarity coefficient, *HU* Hounsfield unit, *ICC* intraclass correlation coefficient, *IPFD* intra-pancreatic fat deposition, *MRI* magnetic resonance imaging, *PCC* Pearson correlation coefficient, *PDFF* proton density fat fraction, *PPV* positive predictive value, *RAVD* relative average volume difference, *ROI* region of interest

^a^ Data from the most experienced radiologist (5 years of experience) are presented from the study by Lin et al [15]

^b^ Data from the most experienced radiologist (14 years of experience) are presented from the study by Yang et al [16]

### Clinical domain

Eight studies in the clinical domain applied AI-based models to quantify IPFD in disease states (T2DM [18, 19, 21, 23–25], acute pancreatitis and pancreatic cancer [19], as well as COVID-19 pneumonia [22]) or in regard to physiological outcomes (prevalence of high IPFD [19], ageing [19–21], and sex differences in IPFD [17, 19, 21, 23]).

#### Disease states

Five studies reported on IPFD in the presence and absence of T2DM [18, 19, 21, 24, 25], one of them additionally looked at IPFD in various parts of the pancreas [18]. One study looked at changes in IPFD over time in individuals with T2DM [23]. All studies that investigated the association between IPFD and T2DM found a significant association between the two. The cross-sectional study by Yang et al [25] showed that the pancreatic fat fraction was significantly higher in individuals with T2DM than in normoglycaemic individuals (*p* < 0.01). The other cross-sectional studies also reported similar associations. Liu et al [21] reported a mean pancreatic fat percentage of 10.4% in their cohort and found a significant association with T2DM (*p* < 0.001), but not with type 1 diabetes mellitus (*p* = 0.241). Bagur et al [18] compared the proton density fat fraction (PDFF) in T2DM individuals with high BMI individuals without T2DM. The authors found that only the body of the pancreas had a significantly higher PDFF (12.8% and 11.7% respectively, *p* < 0.05). In contrast, there were no statistically significant differences between the two groups in terms of IPFD in other regions of the pancreas (head, tail) and whole pancreas (all *p* > 0.05). When comparing T2DM individuals with low BMI versus those without T2DM, the authors showed statistically significant differences in PDFF across not only the whole pancreas, but also in the head, body and tail of the pancreas (all *p* < 0.05). The CT study by Tallam et al [24] demonstrated that individuals with T2DM had lower pancreatic CT attenuation (indicative of higher IPFD) than individuals without T2DM (mean of 18.7 HU and 30.0 HU, respectively, *p* < 0.001). Two studies investigating diabetes were longitudinal cohort studies [19, 23]. The study by Dong et al [19] had a mean follow-up period of 4.6 years and found that individuals with high IPFD had a 22% higher risk of T2DM (*p* < 0.001). The study by Whitcher et al [23] was another longitudinal cohort study, and the authors investigated changes in IPFD over a mean follow-up period of 2.2 years in individuals with previously diagnosed chronic diseases (including but not limited to T2DM). The study showed that chronic diseases did not result in a statistically significant change in IPFD (*p* > 0.05) during the study period.

While diabetes was the most commonly investigated disease in the clinical domain of included studies, the study by Dong et al [19] also explored the associations of high IPFD with acute pancreatitis and pancreatic cancer. High IPFD was found to be significantly associated with an increased risk of either disease (HR 1.513, *p* = 0.001 and HR 1.365, *p* = 0.017, respectively). Tanabe et al [22] focused on the association between IPFD and the severity of COVID-19 pneumonia. The authors determined the CT fat volume fraction and found that it had a significant positive correlation with the lung severity score (*p* < 0.01).

#### Physiological outcomes

The study by Dong et al [19] found a 17.9% prevalence of high IPFD (defined as the 95th percentile age- and sex-specific upper limit of IPFD) in the UKBB. Furthermore, they noted an increasing prevalence of high IPFD with age in both men and women individually and combined (*p*-trend < 0.001), with a prevalence of 16.4% in the 45–50 year age group and 25.2% in the 81–85 year age group. The study by Gatidis et al [20] combined data from the National German Cohort (NAKO) and the UKBB to assess age-related changes in pancreas PDFF across a wider age range (20–80 years). The authors reported on a non-linear increase in IPFD with age. In addition, the study by Liu et al [21] noted a statistically significant increase in IPFD with age (*p* < 0.001). While these studies looked at IPFD across different age groups cross-sectionally, the study by Whitcher et al [23] investigated changes in mean PDFF longitudinally. Although the results did not reach statistical significance, the authors found an 11.6% increase in PDFF (*p* = 0.0652) among men, as compared with a 10.4% increase in PDFF (*p* = 0.3441) among women.

The studies by Liu et al [21] and Whitcher et al [23]—both based on the UKBB—also quantified IPFD overall and separately in men and women. Both studies yielded similar findings, with the study by Liu et al [21] reporting a mean pancreatic fat percentage of 12.6 ± 8.5% and the study by Whitcher et al [23] reporting a median PDFF of 12.2 ± 8.3% in men. For women, the findings were 8.3 ± 6.7% and 8.0 ± 5.9%, respectively. In terms of combined IPFD, the study by Liu et al [21] reported a mean of 10.4 ± 7.9% whereas the study by Whitcher et al [23] reported a median total PDFF of 10.1 ± 7.5%. Although the study by Zhang et al [17] had a smaller sample (*n* = 22) in their testing set, they reported a similar mean fat volume fraction of 12.7 ± 9.8%.

## Discussion

This is the first systematic review to have summarised data related to the current state of IPFD quantification with the use of AI. The present review included a total of 12 studies covering over 50,000 participants from around the world. Our pooled results showed that the performance of AI-based models in regard to pancreas segmentation in studies quantifying IPFD reached a mean weighted DSC of 82.3% (95% confidence interval from 73.5 to 91.1%), indicating the non-negligible dissimilarity between manual and AI-based approaches. This finding builds on the results of our 2019 systematic review and meta-analysis that yielded a mean weighted DSC of 74.4% (95% confidence interval from 70.9 to 77.8%) for AI-based models of pancreas segmentation alone (i.e., without quantification of IPFD) [9]. It is reasonable to suggest that the pixel-level precision of AI-driven determination of pancreas boundaries—the single most important step in automated IPFD quantification—has generally improved over the past 5 years or so, but in a rather piecemeal fashion, as DCS is still very far from 100%.

Novel insights were gained from the studies under the developmental domain in the present systematic review. AI-based models are nowadays commonly integrated into the software of many CT and MRI scanners, enabling automated segmentation and quantification of fat in the liver. This is largely because liver segmentation tends to be much more accurate, often achieving nearly perfect DSC. For instance, a 2024 study reported a DSC of 98% for liver segmentation [26]. The liver’s larger, more uniform shape and consistent position within the abdomen make it markedly easier to segment the liver compared with the pancreas. In contrast, pancreas segmentation presents greater challenges, leading to lower DSC values. As a result, AI-based models for IPFD quantification are yet to be ready for widespread clinical application. Despite these challenges, Lin et al [15] demonstrated that AI models, particularly nnU-Net, provided more consistent and reliable IPFD quantification, with a high ICC of 0.936 for test-retest reliability. In comparison, radiologists achieved a test-retest reliability ranging from 0.641 to 0.737. The high levels of consistency and reduced inter-rater variability in the employed nnU-Net model suggest that there is potential for AI-based models to provide more consistent quantification of IPFD as compared with manual means. This becomes especially important in the clinical domain, where AI models for IPFD quantification are employed to evaluate both diseased and physiological states [1, 2].

The most frequently used AI-based model in the clinical domain was U-Net [18, 21, 24, 25], with DSC ranging from 69.0% [24] to 91.2% [25]. U-Net is a form of CNN architecture that was developed for biomedical image segmentation. V-Net extends the concepts of U-Net to directly handle medical imaging data using 3D convolutional layers. One study applied V-Net and U-Net models to pancreatic segmentation (without IPFD quantification) on the same CT dataset and found accurate and robust segmentation across both models [27]. The authors noted, though, that U-Net performed segmentation with much fewer input parameters and was able to be trained four times faster than V-Net [27]. The more recent studies (from 2023 to 2024) in the present systematic review [15, 16, 19, 20] used nnU-Net or nnTransfer model [24], which yielded DSC ranging from 80.7% [16] to 93.7% [17]. nnU-Net, short for ‘no-new-U-Net’, is an extension of U-Net architecture that automatedly configures its architecture, preprocessing, and training procedures based on the dataset’s characteristics. nnU-Net automates many of the manual decisions when using U-Net, such as deciding how deep the network should be, how large the input images should be, and how the data should be preprocessed [9, 19]. This automation simplifies the use of nnU-Net, increasing its accessibility. nnTransfer, short for ‘nonisomorphic transfer learning net’, is a novel 3D segmentation model that employs a generative model structure for self-supervision, enabling the network to learn image attributes from unlabelled data [9, 17]. The choice of imaging modality may affect the performance of AI-based models. While nnTransfer model had the highest DSC among CT studies, nnU-Net had the highest DSC among MRI studies. Further, DSCs were more consistent across the MRI studies, ranging from 84.2% [14] to 91.6% [15]. This is as compared with DSCs in the CT studies that ranged from 69.0% [24] to 93.7% [17]. While studies included in the present review did not evaluate the performance of a single AI model on both CT and MRI scans, a 2025 study focusing solely on AI pancreatic segmentation (without IPFD quantification) developed an AI model combining nnU-Net with a linear self-attention layer [28]. This model was shown to achieve a similar (and reasonably good) performance on both CT and MRI scans [28].

Novel insights were also gained from the studies under the clinical domain. Precise quantification of IPFD can enable early detection and, hence, better management of the diseases caused by high IPFD such as T2DM, pancreatic cancer, and pancreatitis [2, 29, 30]—all of which are diseases that significantly impact patient morbidity and mortality, as well as contribute to significant healthcare costs. Therefore, enhancing the accuracy and reliability of AI-based models will directly contribute to improved diagnostic capabilities, facilitating early intervention, and potentially mitigating the progression of the disease implicated by high IPFD—an idea central to the PANDORA hypothesis [2]. Additionally, while the link between high IPFD and T2DM is well acknowledged in the literature [19], the studies in the present systematic review replicated these findings using AI-based models. AI provided streamlined and effective insights into diseases of the pancreas, allowing the body of evidence on the association between IPFD and T2DM to be expanded. Beyond pancreatic disease, IPFD quantification has potential applications in other conditions. For instance, high IPFD has been associated with cardiac complications [31], Alzheimer’s disease [32], diseases of iron overload [33], hypothyroidism [34], obstructive sleep apnoea [35], polycystic ovarian syndrome [36], and more severe cases of COVID-19 pneumonia [22]. This suggests a possible role for accurate IPFD quantification in prognostic evaluations beyond endocrine and exocrine diseases of the pancreas. The broad applicability of IPFD measurements highlights the value of advancing AI-based models for automated IPFD quantification with a view to enhancing research and clinical practice across a range of health conditions.

While AI holds great promise for IPFD quantification, several factors constrain its widespread application at this point in time. First, creating accurate, fully annotated ground truths for developing AI-based models is time-consuming, resource-intensive, and susceptible to human error [15, 24, 28]. Adopting weakly supervised learning approaches, where data annotation is kept minimal and essential, may expedite the training process. Furthermore, employing consensus-based annotation (where multiple experienced radiologists label the ground truths) may help minimise human error. It is reassuring that some studies in the present systematic review have already done so [16, 25]. Second, an AI-based model may occasionally miss small or subtle features (in particular, in complex but small organs such as the pancreas), leading to misclassification or inaccurate IPFD quantification. Guiding the AI using attention mechanisms to focus on critical aspects (such as boundaries of the pancreas and anatomical landmarks) in the developmental phase could help to eliminate this shortcoming. Another limitation is that the use of only one imaging modality may limit AI accuracy, as different modalities have certain strengths and weaknesses. Exploring multimodal fusion, where multiple imaging modalities are combined during training, has the potential to enhance IPFD quantification. Last, domain shifts in pancreas segmentation performance could be substantial. For example, a 2025 MRI study found that the DCS decreased by up to 10% upon external validation [28].

The present systematic review has a number of limitations. First, many included studies relied on the UKBB, which only includes middle-aged and elderly people and has virtually no ethnic diversity. In addition, most studies lacked long-term follow-up—a key aspect in assessing the real-world utility of AI-based models across diverse clinical settings and imaging modalities [37, 38]. These aspects need to be addressed in future research. Hospitals often cannot share patient images with one another due to privacy concerns, which can limit the diversity and volume of training data as well as generalisability. Incorporation of federated learning across multiple hospitals and institutions to train a single AI-based model collaboratively (without directly sharing patient data) may help to overcome this limitation. Models would be trained locally, and only the insights gained would be shared, rather than the raw data. Second, the studies in the developmental domain tested their models on different datasets, making direct comparisons between models prone to bias. Future AI-based IPFD quantification studies should aim to assess the performance of different models on the same dataset. Third, AI-based models were reported inconsistently in the included studies. IPFD quantification studies using AI would benefit from reporting standardised performance measures for both segmentation and quantification, including statistical components such as mean, standard deviation and *p*-value. Fourth, of the studies included in the meta-analysis, the overwhelming majority were CT studies. This highlights the need for additional MRI studies to further assess the performance of AI-based models in clinical settings [39]. Last, the included studies lacked information on the computational cost of deploying AI-based models and quantifying IPFD. One study compared various AI-based models of brain segmentation and found that, while 3D segmentation models were faster to train and deploy than 2.5D and 2D approaches, 3D models required substantially more computational memory [40]. Future studies should aim to include information concerning computational costs such as runtime, memory requirements, and inference speeds. This would ensure that AI-based models are not only accurate but also that their real-world clinical application is feasible.

In conclusion, IPFD quantification aids in the diagnosis and prognosis of both pancreatic and non-pancreatic disease. While it is most commonly and ethically done using modern imaging techniques (such as MRI and CT), IPFD quantification is currently overlooked compared with other abdominal organs (such as the liver). The few studies that did investigate IPFD quantification using AI-based models tended to yield suboptimal results. Future research should focus on compiling large, diverse imaging datasets that include longitudinal data to develop and validate robust AI-based models for IPFD quantification.

## Supplementary information

ELECTRONIC SUPPLEMENTARY MATERIAL

## Abbreviations

AI: Artificial intelligence
BMI: Body mass index
CPU: Central processing unit
CT: Computed tomography
DL: Deep learning
DSC: Dice similarity coefficient
GPU: Graphics processing unit
HU: Hounsfield unit
ICC: Intraclass correlation coefficient
IPFD: Intra-pancreatic fat deposition
IQR: Interquartile range
MRI: Magnetic resonance imaging
NAKO: German National Cohort
PCC: Pearson correlation coefficient
PDFF: Proton density fat fraction
ROI: Region of interest
SD: Standard deviation
T2DM: Type 2 diabetes mellitus
UKBB: United Kingdom Biobank
