# Supplementary material for: Artificial intelligence-based models for quantification of intra-pancreatic fat deposition and their clinical relevance: a systematic review of imaging studies
Source: Eur Radiol. 2025 Jul 19;36(1):627–41. doi: 10.1007/s00330-025-11808-6 (PMC12711924; doi:10.1007/s00330-025-11808-6)
Supplement: Supplementary file 1 — ELECTRONIC SUPPLEMENTARY MATERIAL [file 330_2025_11808_MOESM1_ESM.pdf]

Artificial intelligence-based models for quantification of intra-pancreatic fat deposition and their clinical relevance: a systematic review of imaging studies

ELECTRONIC SUPPLEMENTARY MATERIAL

Supplementary Table 1. Methodological quality assessment of the included studies

Study ID	JBI appraisal score[a]										
**Longitudinal studies**	**1**	**2**	**3**	**4**	**5**	**6**	**7**	**8**	**9**	**10**	**11**
Dong et al. [19]	Y	Y	Y	Y	Y	Y	Y	Y	U	U	Y
Whitcher et al. [23]	Y	Y	Y	Y	Y	Y	Y	Y	Y	U	Y
**Cross-sectional studies**				**1**	**2**	**3**	**4**	**5**	**6**	**7**	**8**
Basty et al. [14]				Y	Y	Y	Y	N	N	Y	N
Gatidis et al. [20]				Y	Y	Y	Y	Y	Y	Y	Y
Lin et al. [15]				Y	Y	Y	Y	Y	Y	Y	Y
Liu et al. [21]				Y	Y	Y	Y	Y	Y	Y	Y
Tallam et al. [24]				Y	Y	Y	Y	Y	Y	Y	Y
Tanabe et al. [22]				Y	Y	Y	Y	Y	Y	Y	Y
Triay Bagur et al. [18]				Y	Y	Y	Y	Y	Y	Y	Y
Yang et al. [25]				Y	Y	Y	Y	Y	Y	Y	Y
Yang et al. [16]				Y	Y	Y	Y	Y	Y	Y	Y

Zhang et al. [17]	Y	Y	Y	Y	N	N	Y	Y
Abbreviations: Y, yes; N, no; U, unclear.<br><br>Footnote: The JBI critical appraisal checklist for longitudinal studies consisted of 11 questions whereas there were eight questions for cross-sectional studies.								
